# Commentary: The histone demethylase Phf2 acts as a molecular checkpoint to prevent NAFLD progression during obesity

**DOI:** 10.3389/fgene.2018.00443

**Published:** 2018-10-16

**Authors:** Barbara Barbaro, Ilaria Romito, Anna Alisi

**Affiliations:** Research Unit of Molecular Genetics of Complex Phenotypes, Bambino Gesu' Children Hospital, Rome, Italy

**Keywords:** NAFLD (non-alcoholic fatty liver disease), NASH (non-alcoholic steatohepatitis), Phf2, histone modification, epigenetics (MeSH)

Non-alcoholic fatty liver disease (NAFLD) is a multi-faceted and multi-factorial hepatic disease, characterized by different patterns of histological damage, which range from hepatosteatosis to non-alcoholic steatohepatitis (NASH). NASH is histologically characterized by hepatosteatosis, hepatocellular ballooning, lobular inflammation, and fibrosis, which may progress to cirrhosis and, potentially, end-stage liver disease and hepatocellular carcinoma (Yeh and Brunt, 2014).

It is widely accepted that NAFLD could be a heritable disease in which genetic variants, including those of *PNPLA3, TM6SF2*, and *MBOAT7* genes, and epigenetic drivers linked to over-nutrition closely interact to determine the disease phenotype and its progression (Eslam et al., 2018). Thus, epigenetic modifications, including chromatin remodeling, histone modifications, DNA methylation and non-coding RNAs, make a major contribution in determining the NAFLD onset and progression (Eslam et al., 2018). A genome-wide integrated methylome/transcriptome analysis has demonstrated that genes involved in the methylation process, inflammation, and fibrogenesis showed a stage-dependent regulation, suggesting that epigenetic changes are involved in the progression of NAFLD (Murphy et al., 2013).

The recently-emerged fact that nutritional epigenetics may explain the gene-diet interactions, further elucidating the modulatory role of nutrition in diseases, is noteworthy. In line, NAFLD may exhibit a different pattern and level of severity of tissue damage in function of the excess of specific nutrients in the diet. Several studies have showed that histone modifications, such as acetylation and methylation, may contribute to diverse diet-induced metabolic dysfunctions (Murphy et al., 2013). However, a comprehensive analysis of the histone modifications and their dynamic changes in NAFLD is infrequent, hampering our understanding of the role of diet-related epigenetic mechanisms in the development and progression of this disease.

In this regard, a recent article published in Nature Communication (Bricambert et al., 2018) demonstrated that the histone demethylase Plant homeodomain finger 2 (Phf2) protects liver from NAFLD progression. The authors found that an over-expression/induction of Phf2 protected the liver from lipotoxicity and oxidative stress in models of a high-fat/high-sucrose diet (HF/HSD) dependent NAFLD. The proposed mechanism was that a Phf2 over-expression regulated the promoters of several genes including PKLR, ACLY, ACCA, FASN, SCD1, ELOVL6, TXNIP, RGS16, PNPLA3, and FGF21 by facilitating H3K9me2 histone demethylation at a carbohydrate-responsive element binding protein (ChREBP). Previous studies investigated the Phf2 role in metabolism (Baba et al., 2011; Okuno et al., 2013). Baba et al. (2011) suggested that Phf2 is inactive by itself, but becomes an active H3K9Me2 demethylase through PKA-mediated phosphorylation, with a major role in the induction of gluconeogenic genes (Baba et al., 2011). More recently, Okuno et al. (2013) demonstrated a role for Phf2, in the regulation of adipogenesis. Specifically, the authors demonstrated in Phf2-knockout mice, that Phf2 potentiates adipogenesis through an interaction with the transcription factor, the CCAAT/enhancer-binding protein alpha, indicating Phf2 as a potential new therapeutic target in the treatment of obesity and the metabolic syndrome (Okuno et al., 2013).

Importantly, the article by Bricambert et al. (2018) fits well into the deepening of the role of Phf2 in metabolic diseases. Specifically, in a mouse model of HF/HSD-induced NAFLD, the authors demonstrated that even by the triggering of diet-induced hepatic steatosis, Phf2 concomitantly reduced lipotoxicity by increasing the production of mono-unsaturated fatty acids (MUFA), thus enhancing the MUFA/saturated fatty acids (SFA) ratio. Phf2 over-expression also reduced oxidative stress by improving the activity of the NF-E2-related factor 2 (Nrf2). Findings by Bricambert et al. (2018) highlighted that both Phf2 and ChREBP are functionally co-recruited to the Nrf2 promoter in response to glucose, thus increasing the Nrf2 expression and its activity on the genes of specific targets. Indeed, the authors reported that the Phf2-mediated activation of Nrf2, redirects glucose toward the pentose phosphate pathway and glutathione biosynthesis, defending the liver from the accumulation of reactive oxygen species and consequently, oxidative stress.

The Phf2-dependent reduction of oxidative stress rebounded like a cascade on several other genes that control hepatic inflammation and fibrosis, implying a consequent great reduction of their expression. Previous studies highlighted the crucial role of Nrf2 and its signaling pathways in protecting hepatic cells from oxidative damage during the development of common chronic liver diseases, indicating Nrf2 as a therapeutic target (Meakin et al., 2014; Sharma et al., 2017). Meakin et al. (2014) showed that Nrf2 deficiency made the mice more sensitive to develop NASH when placed on an HFD, by the induction of lipogenesis genes and the suppression of β-oxidation genes. More recently, the same group of authors (Sharma et al., 2017) demonstrated that a potent pharmacologic activator of Nrf2 (TBE-31) ameliorated experimental NASH and liver fibrosis reducing insulin resistance, suppressing hepatic steatosis, and inhibiting inflammatory response and oxidative stress. However, the existence of some adverse effects of Nrf2 activation cannot be ignored. In fact, among others, a phase 3 clinical trial, evaluating the Nrf2-pathway activator Bardoxolone methyl, in type 2 diabetes and stage 4 chronic kidney disease, has been interrupted as it did not reduce the risk of end-stage renal disease/death from cardiovascular causes (de Zeeuw et al., 2013). These findings are important, considering that Phf2 acts on Nrf2. Moreover, the fact that a Phf2 over-expression promotes hepatic fat accumulation should not be underestimated.

Several prevailing drugs, evaluated in clinical trials on NASH patients have been proven to slightly improve only one feature of the hepatic disease without exacerbating the others (Townsend and Newsome, 2017).

On the other hand, the Phf2-dependent increase of hepatosteatosis is a no-progressive feature in the pre-clinical study by Bricambert et al. (2018), suggesting the possibility to employ Phf2 pharmaceutical targeting as a therapeutic approach.

Hopefully it might be hypothesized that the anti-fibrogenic/inflammatory therapies based on Phf2 activation could be an option, in combination with diet, physical exercise and nutritional supplements that are recognized as anti-steatotic (Figure 1) (Romero-Gómez et al., 2017).

**Figure 1:**
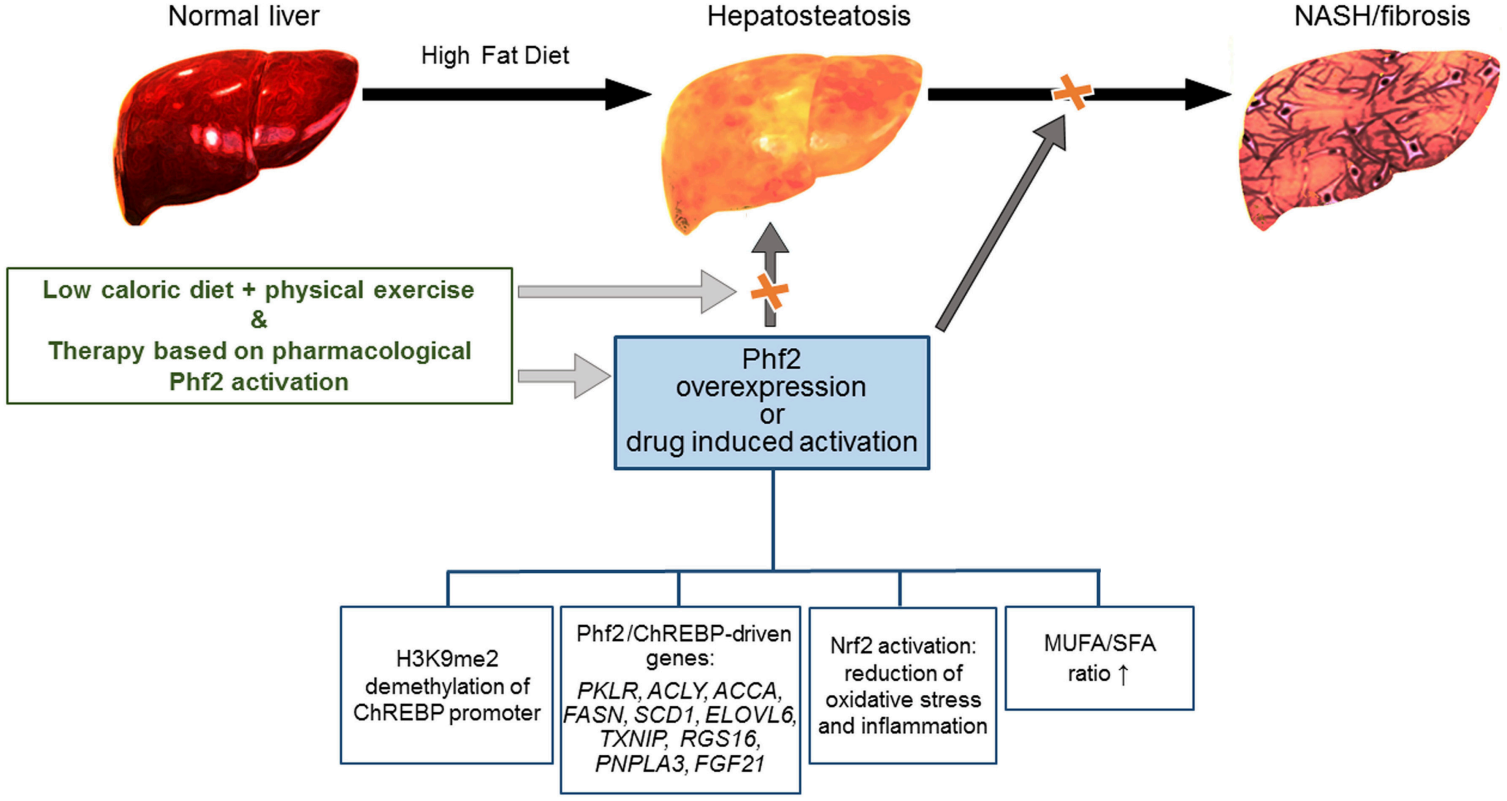
Schematic representation of the Phf2 action in NAFLD. Phf2 activation, through H3K9me2 demethylation at specific ChREBP-regulated gene promoters, increases MUFA synthesis and provides protection from the progression of hepatosteatosis toward NASH. Therapy based on Phf2, in combination with a low-caloric diet and physical exercise could be an active therapeutic approach against NAFLD.

In conclusion, the article by Bricambert et al. (2018) provides a great contribution to the large landscape of epigenetics, and could serve as another brick in the field dealing with the use of epigenetic drugs as one of the multiple treatments to be included in multi-target therapeutic strategies in our fight against the development and progression of NAFLD.

## Author contributions

BB, IR, and AA wrote the text. BB and IR designed the figure. AA critically revised the final manuscript. All authors have approved the manuscript.

### Conflict of interest statement

The authors declare that the research was conducted in the absence of any commercial or financial relationships that could be construed as a potential conflict of interest.
